# Is there an association between the use of complementary medicine and vaccine uptake: results of a pilot study

**DOI:** 10.1186/s13104-018-3323-8

**Published:** 2018-04-02

**Authors:** Jane E. Frawley, Erica McIntyre, Jon Wardle, Debra Jackson

**Affiliations:** 1 Australian Centre for Public and Population Health Research, Faculty of Health, University of Technology Sydney, Level 8, 235 Jones St, PO Box 123, Ultimo, NSW 2007 Australia; 2 Australian Research Centre in Complementary and Integrative Medicine (ARCCIM), Faculty of Health, University of Technology Sydney, Ultimo, NSW 2007 Australia; 3 Oxford Institute of Nursing & Allied Health Research, Faculty of Health & Life Sciences, Oxford Brookes University, Harcourt Hill Campus, Oxford, OX2 9AT UK

**Keywords:** Immunisation, Vaccination, Complementary medicine, Health service research, Paediatric

## Abstract

**Objective:**

Despite the incredible success of paediatric immunisation, support is not universal. It has been suggested that complementary medicine practitioners enable vaccine rejection and his study aims to explore the relationship between complementary medicine use and paediatric vaccination. A total of 149 Australian parents were recruited via a parenting website and Facebook groups to complete an online questionnaire.

**Results:**

The majority of parents (66.4%) stated that their children’s vaccination status was up-to-date. Vaccination status was associated with parental education, area of residence, income, private health insurance, and having a Health Care Card (p < 0.05). Children’s vaccinations were more likely to be up-to-date if they had consulted a general practitioner in the previous 12 months (OR 21.75; p < 0.001), and less likely to be up-to-date if they had consulted a complementary medicine practitioner (OR 0.10; p < 0.001) in the same period. Concerns about vaccine safety and efficacy were the most common reasons for a child’s immunisation status not being up-to-date. These findings highlight an interface between lower vaccine uptake and visits to complementary medicine practitioners. These results emphasise the need to examine the routine paediatric care practices of complementary medicine practitioners as a crucial piece of the puzzle in understanding vaccine rejection.

**Electronic supplementary material:**

The online version of this article (10.1186/s13104-018-3323-8) contains supplementary material, which is available to authorized users.

## Introduction

Childhood immunisation has successfully reduced the incidence of infectious disease and childhood mortality worldwide, and the high rate of childhood vaccination coverage in most high-income countries indicates that paediatric vaccination remains a widely accepted public health measure [1]. However, support for paediatric vaccination is not universal, with vaccine rejection viewed as an emerging international public health problem [2]. Complementary medicine (CM)—a diverse group of health care practices not considered part of the conventional medical curriculum [3]—is one phenomenon that is portrayed as a possible enabler in vaccine refusal [4].

In Australia, as with many other nations, vaccination rates are generally high enough to infer herd immunity [5]. However, there is concern that the long-term success of the childhood immunisation program is vulnerable to the growing threat of vaccine rejection and anti-vaccination sentiment [6]. Recently, significant attention has been drawn to the issue of vaccine hesitancy and its perceived link with the anti-vaccination movement and CM [7, 8]. Despite high levels of CM utilisation in Australia [9] and highly publicised concerns about the impact of CM on vaccine rejection, little is currently known about the relationship between CM use and paediatric vaccination in Australia. As health consumers increasingly turn to social networks and other Internet resources to inform their decision making on health issues such as vaccination [10], it is important to investigate this phenomenon among active users of Internet forums. This study aims to address this critical research gap by investigating the association between CM use and paediatric vaccination among users of a popular Australian parenting website and online parenting groups.

## Main text

### Methods

#### Participants

We conducted an online survey of Australian parents with children up 18 years of age. The survey was posted on one of Australia’s largest national parenting websites (BubHub) as well as Facebook parenting groups.

#### Questionnaire

The questionnaire included both closed and open-ended questions, focusing on children’s conventional health service use, visits to CM practitioners, CM product use, and vaccination status (see Additional file 1). The variables in the questionnaire were adapted from previous studies in similar population samples [11, 12], following a review of the literature. They were refined following discussions with parents and content experts in the research team. Information about parent’s gender, marital status, age, income, employment and level of education was collected along with their attitudes to vaccination. Participants were also asked to classify their place of residence as either urban (capital city or major metropolitan centre with a population > 100,000) or non-urban (population < 100,000) and if they had private health insurance or a Health Care Card.

Parents were asked about the health services they had visited for their children’s health needs in the previous 12 months. These health services included conventional medical services [general practitioner (GP), paediatrician], and CM practitioners including a naturopath/herbalist, nutritionist, osteopath, chiropractor, massage therapist, traditional Chinese medicine practitioner or homoeopath. Parents were also asked if their child had used a CM product in the previous 12 months, including herbal medicine, vitamin and mineral supplement, aromatherapy or homoeopathy. Additionally, parents were asked if their children’s vaccination status was up to date according to the Australian vaccination schedule. If their child’s vaccinations were not up to date, parents were asked further about their motivations and attitudes towards vaccination.

#### Statistical analysis

The characteristics of parents choosing to vaccinate or not vaccinate their children were investigated, and relationships were determined using a Chi square analysis. Identification of significant covariates was also determined through univariate logistic regression between all possible predictors (i.e. the demographic and health care variables) and children’s vaccination status. All the demographic and health service utilisation variables were entered into a model, and then a stepwise backward elimination process was employed, using a likelihood ratio test, to produce the most parsimonious model. Adjusted odds ratios and 95% confidence intervals were calculated and statistical significance was set at p < 0.05. All analyses were conducted using statistical program STATA 14.1 (StataCorp LP, College Station, TX, USA).

### Results

A total of 149 parents responded to the online study of which 97.3% were female (as shown in Table 1). Parents were more likely to have one (n = 62, 41.3%) or two (n = 73, 48.7%) children, aged between 2 and 5 years (n = 78, 52.0%). The majority of respondents were aged between 35 and 44 years (n = 81, 54.4%), married or living with a partner (n = 135, 90.6%), and were financially comfortable (n = 84, 56.8%). Parents were more likely to have private health insurance for both hospital and extras (n = 78, 54.9%), less likely to have a Health Care Card (n = 116, 78.9%) and many had attained a degree or postgraduate degree as their highest education qualification (n = 117, 78.5%).

**Table 1:** Demographic features and health care visits with vaccine status

	Vaccination up to date (n = 99; 66.4%)	Vaccination not up to date (n = 48; 33.6%)	p value
Visit to conventional health practitioners in previous 12 months			
General practitioner	95 (72.0)	37 (28.0)	< 0.001
Paediatrician	31 (70.4)	13 (29.6)	0.548
Community nurse	33 (71.7)	14 (28.3)	0.443
Visit to complementary medicine practitioners in previous 12 months			
Naturopath/herbalist	19 (42.2)	26 (57.8)	< 0.001
Nutritionist	6 (60.0)	4 (40.0)	0.608
Osteopath	10 (45.5)	12 (54.5)	0.018
Chiropractor	14 (51.9)	13 (48.1)	0.057
Traditional Chinese medicine practitioner	4 (33.3)	8 (66.7)	0.009
Homoeopath	1 (5.9)	16 (94.1)	< 0.001
Massage therapist	4 (66.7)	2 (33.3)	0.791
Use of complementary medicine			
Herbal medicine	25 (43.9)	32 (56.1)	< 0.001
Vitamins/minerals	45 (57.7)	33 (42.3)	0.002
Essential oils	19 (43.2)	25 (56.8)	< 0.001
Homoeopathic	19 (39.6)	29 (60.4)	< 0.001
Demographic features			
Gender			0.713
Female	96 (66.2)	49 (33.8)	
Male	3 (75.0)	1 (25.0)	
Age			0.138
18–24	0	0	
25–34	32	15	
35–44	57	24	
45–54	10	11	
55+	0	0	
Area of residence			0.003
Urban	86 (72.7)	33 (27.73)	
Rural	13 (43.3)	17 (56.7)	
Highest level of education			0.010
Up to year 12	7 (77.8)	2 (22.2)	
Trade/apprenticeship	9 (39.1)	14 (60.9)	
University degree	83 (71.0)	34 (29.1)	
Manage on available income			0.035
Difficult all the time	9 (47.4)	10 (52.6)	
Difficult sometimes	27 (60.0)	18 (40.0)	
Not too bad	63 (75.0)	21 (25.0)	
Relationship status			0.006
Never	0 (0.0)	1 (100.0)	
Married/defacto	95 (70.4)	40 (29.6)	
Separated/widowed/divorced	4 (30.8)	9 (69.2)	
Private health insurance			0.002
None	20 (45.5)	24 (54.6)	
Yes, hospital only	13 (86.7)	2 (13.3)	
Yes, extras only	2 (40.0)	3 (60.0)	
Yes, both hospital and extras	58 (74.4)	20 (25.6)	
Health Care Card	14 (45.2)	17 (54.9)	0.005

A total of 66.4% (n = 99) of parents stated their children’s vaccination status was up to date, while 33.6% were not up to date. Chi square analysis (see Table 1) found that vaccination status was more likely to be up to date if the child has visited a GP in the previous 12 months (p < 0.001) and less likely to be up to date if they had visited a naturopath/herbalist (p < 0.001), osteopath (p = 0.018), traditional Chinese medicine practitioner (p = 0.009) or homoeopath (p < 0.001). Vaccination was also less likely if the child had used a herbal medicine (p < 0.001), vitamin/mineral (p = 0.002), aromatherapy (p < 0.001) or homoeopathic product (p < 0.001) in the same period. Associations between vaccination and geographical area of residence (p = 0.003), parental education (p = 0.010), income (p = 0.035), private health insurance (p = 0.002), and having a Health Care Card (p = 0.005) were also found.

The most common reasons that a child’s vaccination was not up to date were: concern about side effects and adverse events (n = 36, 76.6%), the belief that vaccines are not safe (n = 18, 38.3%), or effective (n = 18, 38.3%), wanting their child to receive some vaccines but not others (n = 16, 34.0%) and delaying vaccination in the belief that children receive too many vaccines too early in life (n = 13, 27.7%) (see Table 2).

**Table 2:** Reasons vaccinations not up to date

Total number of responders = 47	(%)
I am concerned about side effects and adverse events related to childhood vaccinations	36 (76.6)
I don’t believe vaccines are safe	18 (38.3)
I don’t believe vaccines are effective	18 (38.3)
I want my child to receive some vaccines but not others	16 (34.0)
I want to vaccinate my child but wish to delay as they receive too many too soon	13 (27.7)
I don’t believe vaccines are necessary	11 (23.4)
I would like more information about the side-effects and adverse reactions before vaccinating my child	10 (21.3)
My child has a medical exemption	5 (10.6)
I intend to vaccinate my child but haven’t taken him/her yet due to time pressures	3 (6.4)
I intend to vaccinate my child but haven’t taken him/her yet due to transport problems	0 (0.0)

Table 3 shows the results of the logistic regression model. Children’s vaccinations were more likely to be up to date if the child had consulted a GP in the previous 12 months (OR 21.75; CI 4.24, 111.63; p < 0.001), and less likely to be up to date if they had consulted a CM practitioner (OR 0.10; CI 0.04, 0.28; p < 0.001) in the same period. Vaccination was also less likely if the parent had an Australian Health Care Card that entitles the family to further government subsidies for health care, prescription medicines and other government services such as transport (OR 0.15; CI 0.05, 0.43; p < 0.001).

**Table 3:** Logistic regression—likelihood of vaccination

	Adjusted odds ratio	95% CI	p value
Visits to health professionals in last 12 months			
General practitioner	21.75	4.24, 111.63	< 0.001
Complementary medicine practitioner	0.10	0.04, 0.28	< 0.001
Health Care Card	0.15	0.05, 0.43	< 0.001

### Discussion

This pilot study is the first to investigate associations between vaccine rejection and health service utilisation (including CM) in Australian parents who utilise Internet forums and social networks for parenting advice. The majority of parents reported their children’s vaccination status as up-to-date, albeit at a lower rate than the general Australian population. Having an up-to-date vaccination status was associated with seeing a GP in the previous 12 months. This finding is in line with recent research that has found many parents are influenced by information about vaccination received from a GP [13], highlighting the important role that GPs have in discussing the benefits and risks of vaccination.

Our study found reduced uptake of paediatric vaccines was associated with visits to a CM practitioner, which is consistent with a previous critical review [14]. It is possible a parent’s worldview may influence health care choices; for example, post-modern beliefs, rejection of authority, and beliefs about natural remedies have been found to predict positive attitudes to CM [15]. Similar beliefs have been associated with vaccine rejection, such as distrust of pharmaceutical companies, and fear of vaccine side-effects [12], suggesting that some parents who visit CM practitioners may also have concerns about vaccination. Having an unvaccinated or partially vaccinated child has been shown to be an independent predictor of visiting a CM practitioner [14] and these findings suggest that there may be a relationship between CM practitioner use and vaccine uptake, however causality is yet to be determined.

The influence that CM practitioners may have themselves in the association between lower vaccine rates among CM users remains unclear. While CM practitioners have reported significantly heterogenic attitudes generally, studies have found a large proportion of CM practitioners are acquiescent to vaccination, supporting the mainstream public health agenda [14]. It is not possible to determine the intricacies of associations between CM use and vaccination uptake from the current study; therefore, further research is required to determine how CM practitioners are managing clinical discussions about vaccination with their patients. Such research also needs to challenge assumptions about causality and determine whether CM practitioners’ beliefs about vaccination are influencing parental decision-making, or if lower vaccine uptake and CM practitioner use are expressions of parental ideology and health seeking behaviour.

Research from other health practitioner cohorts has found knowledge is a strong determinant of vaccine attitudes and practices [2] and to date, little is known about CM practitioner education, training and knowledge related to vaccination. Chiropractors and naturopathic students have described relying on informal and ad-hoc sources of education [14] and research is required to determine if a need for further education exists for this workforce.

Our pilot study found the greatest concern expressed by parents about vaccination was safety. Over two-thirds of parents with children who were not vaccinated had concerns about side-effects and adverse events occurring as a result of immunisation. In addition, one-third of parents said they do not believe vaccines are safe. Safety concerns are a common theme in the literature describing vaccine hesitancy and refusal, even when such risks remain small [2]. Concerns about the safety of conventional pharmaceuticals have also frequently been associated with CM use [14], suggesting that concerns about the safety of medicines more broadly may be a common factor in both CM use and vaccine rejection.

Lower vaccine uptake was associated with having a Health Care Card (provided to low-income earners by the Australian government, allowing access to cheaper prescription medicines and various other government concessions). The association between having a Health Care Card and lower rates of vaccination is concerning and requires further research to ensure the provision of equitable health care and prevention of vaccine preventable diseases for all Australian children. This finding may also suggest that focusing solely on anti-vaccination sentiment as the primary cause of lower vaccination uptake may obfuscate other potential issues around access and affordability (not limited to medication or consultation costs, but also transport and time off work for visits), particularly in vulnerable communities [16].

### Conclusion

This pilot study suggests that parents who consult CM practitioners for their children’s health care needs are less likely to have children who are fully vaccinated, whereas those who see their GP more regularly are more likely to be fully vaccinated. Factors such as specific beliefs about vaccines related to safety and efficacy and level of education were also associated with vaccine rejection. The critical examination of vaccination advice sought by parents who visit CM practitioners is an important piece of the puzzle that helps to inform this current public health challenge.

## Limitations

- This was a pilot study; therefore, findings need to be interpreted in this context. Larger studies with a more nationally representative sample of parents are needed to confirm the findings reported here.
- A higher proportion of respondents in this sample were CM users, and rates of vaccination were lower than the national average.

## Additional file

**Additional file 1.** Survey_questionnaire.

## Abbreviations

CM: complementary medicine
CI: confidence interval
GP: general practitioner
OR: odds ratio

## References

[1] World Health Organization. 2018. Immunization coverage. http://www.who.int/mediacentre/factsheets/fs378/en/. Accessed 23 Jan 2018.

[2] Dube E, Laberge C, Guay M, Bramadat P, Roy R, Bettinger J (2013). Vaccine hesitancy: an overview. Hum Vaccin Immunother.

[3] Adams (2012). Traditional, complementary and integrative medicine: an international reader.

[4] Ernst E (2001). Rise in popularity of complementary and alternative medicine: reasons and consequences for vaccination. Vaccine.

[5] National Health Performance Authority. 2017. Healthy Communities: Immunisation rates for children in 2015–16. https://www.myhealthycommunities.gov.au/our-reports/immunisation-rates-for-children/june-2017. Accessed 15 June 2017.

[6] Corben P, Leask J (2016). To close the childhood immunization gap, we need a richer understanding of parents’ decision-making. Hum Vaccin Immunother.

[7] Wardle J, Stewart C, Parker M (2013). Jabs and Barbs: ways to address misleading vaccination information using currently available strategies. J Law Med.

[8] Browne M, Thomson P, Rockloff MJ, Pennycook G (2015). Going against the herd: psychological and cultural factors underlying the ‘vaccination confidence gap’. PLoS ONE.

[9] Reid R, Steel A, Wardle J, Trubody A, Adams J (2016). Complementary medicine use by the Australian population: a critical mixed studies systematic review of utilisation, perceptions and factors associated with use. BMC Complement Altern Med.

[10] Stahl JP, Cohen R, Denis F, Gaudelus J, Martinot A, Lery T (2016). The impact of the web and social networks on vaccination. New challenges and opportunities offered to fight against vaccine hesitancy. Medecine et maladies infectieuses.

[11] Larson HJ, Jarrett C, Schulz WS, Chaudhuri M, Zhou Y, Dube E, Schuster M, MacDonald NE, Wilson R (2015). Measuring vaccine hesitancy: the development of a survey tool. Vaccine.

[12] Yaqub O, Castle-Clarke S, Sevdalis N, Chataway J (2014). Attitudes to vaccination: a critical review. Soc Sci Med.

[13] Chow MY, Danchin M, Willaby HW, Pemberton S, Leask J (2017). Parental attitudes, beliefs, behaviours and concerns towards childhood vaccinations in Australia: a national online survey. Aust Fam Phys.

[14] Wardle J, Frawley J, Steel A, Sullivan E (2016). Complementary medicine and childhood immunisation: a critical review. Vaccine.

[15] McIntyre E, Saliba AJ, Wiener KKK, Sarris J (2015). Prevalence and predictors of herbal medicine use in adults experiencing anxiety: a critical review of the literature. Adv Integr Med.

[16] Hardt K, Bonanni P, King S, Santos JI, El-Hodhod M, Zimet GD (2016). Vaccine strategies: optimising outcomes. Vaccine.

