# Supplementary material for: Is there an association between the use of complementary medicine and vaccine uptake: results of a pilot study
Source: BMC Res Notes. 2018 Apr 2;11:217. doi: 10.1186/s13104-018-3323-8 (PMC5880078; doi:10.1186/s13104-018-3323-8)
Supplement: Supplementary file 1 — Additional file 1. Survey_questionnaire. [file 13104_2018_3323_MOESM1_ESM.pdf]

Insert header with crest

1. How many children under the age of 18 are currently in your care?
    a) 1
    b) 2
    c) 3
    d) 4
    e) 5+

2. In the recent 12 months have you taken your child to visit a: (*mark all that apply*)

    a) GP,
    b) Pediatrician,
    c) Community health nurse,

3. In the most recent 12 months have you taken your child to visit a complementary medicine practitioner? (*mark all that apply*).

    a) No,
    b) Naturopath/herbalist,
    c) Nutritionist,
    d) Homeopath,
    e) Osteopath,
    f) Chiropractor,
    g) Massage therapist,
    h) Traditional Chinese medicine practitioner/acupuncturist,
    i) Other?

4. In the most recent 12 months has your child had any of the following complementary medicines and who prescribed them?

	No	Myself or my partner	Doctor	Pediatrician	Other medical specialist	Nurse/ midwife	Complement ary medicine practitioner	Pharmacist	Health food store	Other
Herbal medicine										
Vitamins or minerals										
Homeopathic medicine										
Aromatherapy oil										
Other nutritional supplement (fish oil, probiotics etc)										
Other										

5. Did you fully vaccinate your child (or are all vaccinations up-to-date) according to the Australian vaccination schedule?

    a) Yes,
    b) No

6. If your child's vaccinations are not up to date according to the Australian vaccination schedule please indicate why. (*Choose all that apply*)

    a) I intend to vaccinate my child but haven't taken him/her yet due to time pressures
    b) I intend to vaccinate my child but haven't taken him/her yet due to transport problems
    c) I want my child to receive some vaccines but not others
    d) I want to vaccinate my child but wish to delay as they receive too many too soon
    e) I am concerned about side effects and adverse events related to childhood vaccinations
    f) I don't believe vaccines are safe
    g) I don't believe vaccines are effective
    h) I don't believe vaccines are necessary
    i) My child has a medical exemption
    j) I would like more information about the side-effects and adverse reactions before vaccinating my child

A little about you:

7. Please indicate your gender

    a) Female
    b) Male

8. Please indicate your age

    a) <19
    b) 20-25
    c) 25-30
    d) 31-36
    e) 37-42
    f) 43-48
    g) 48+

9. Would you class your area of residence as: (*choose only one*)

a) Urban (capital city or other metropolitan centre (urban centre population greater than 100,000))
b) Rural/ outer rural (urban centre population up to 99, 000)

10. What is the highest education qualification you have gained?

a) <Year 10 (school certificate or equivalent)
b) Year 12 (higher school certificate or equivalent)
c) Trade/apprenticeship/certificate/diploma
d) Undergraduate degree (Bachelor degree)
e) Postgraduate degree (honors, Graduate Certificate, Graduate Diploma, Master, Doctorate)

11. How do you manage on the income you have available? (*Mark one only*)

a) It is impossible
b) It is difficult all of the time
c) It is difficult some of the time
d) It is not too bad
e) It is easy

12. What is your present marital status?

a) Never Married
b) Married
c) Defacto (opposite sex)
d) Defacto (same sax)
e) Separated
f) Divorced
g) Widowed

13. Do you currently have a health care card?
This is a card that entitles you to discounts and assistance with medical expenses. This is not the same as a Medicare care. (*Mark one only*)

a) Yes
b) No

14. Do you currently have private health insurance that includes cover for your children? (*Mark one only*)

a) No
b) Yes, just hospital cover
c) Yes, hospital and extras
d) Yes, just cover for extras

Thank-you for your participation
